# Prognostic Value of Tumor-Infiltrating Lymphocytes Differs Depending on Lymphocyte Subsets in Esophageal Squamous Cell Carcinoma: An Updated Meta-Analysis

**DOI:** 10.3389/fonc.2020.00614

**Published:** 2020-04-28

**Authors:** Jiatao Hao, Meng Li, Taohong Zhang, Hui Yu, Ying Liu, Yan Xue, Ruifang An, Shuai Wang

**Affiliations:** ^1^Department of Obstetrics and Gynecology, The First Affiliated Hospital of Xi'an Jiaotong University, Xi'an, China; ^2^General Department, Zhongshan Hospital of Fudan University, Shanghai, China; ^3^Neurology Department, Baoji Central Hospital, Baoji, China; ^4^Department of Thoracic Surgery, Zhongshan Hospital of Fudan University, Shanghai, China

**Keywords:** esophageal squamous cell carcinoma, tumor-infiltrating lymphocytes, lymphocyte subsets, prognosis, meta-analysis

## Abstract

**Background:** Tumor-infiltrating lymphocytes (TILs) play a role in the anti-tumor immune response, and are often found in esophageal squamous cell carcinoma (ESCC).

**Methods:** We performed a systematic review and meta-analysis, aiming to establish pooled estimates for survival outcomes of TILs based on their abundance and infiltrating location. A literature search of PubMed/Medline, Embase, Web of Science and the Cochrane Library was conducted. Studies that investigated the prognostic significance of generalized, CD8+, CD4+, FoxP3+, CD3+, and CD45O+ TILs in ESCC patients were included.

**Results:** In pooled analysis, generalized TILs infiltrating the entire tumor mass were positively associated with disease-free survival (DFS), with a univariate-related hazard ratio (HR) of 0.630 [95% confidence interval (CI) 0.415–0.955], and also positively associated with overall survival (OS), with a univariate-related HR of 0.586 (0.447–0.770) and a multivariate-related HR of 0.621 (0.439–0.878). The pan-tumor, intra-tumor and peri-tumor CD8+ TILs had a favorable effect on OS, with univariate-related HRs of 0.733 (0.555–0.968), 0.797 (0.660–0.962), and 0.776 (0.635–0.948), respectively. Similar results were observed in CD8+ TILs that infiltrated the whole tumor mass, with a multivariate-related HR of 0.705 (0.524–0.947). CD4+, FoxP3+, CD3+, and CD45O+ TILs were not linked to DFS or OS. Subtypes and spatial locations of TILs seemed to influence study outcomes.

**Conclusions:** Experimental and analytical methods of future studies should be carefully designed to avoid overestimating the effect of TILs on prognosis. Our meta-analysis confirms the prognostic efficacy of generalized TILs and CD8+ TILs in esophageal squamous cell carcinoma (ESCC) patients.

## Introduction

Esophageal squamous cell carcinoma (ESCC) is one of the deadliest malignancies (1). The 5-year survival rate is ~20%, largely due to late diagnosis and propensity for metastasis. Therapeutic modalities such as surgical or endoscopic resection and chemoradiation have contributed to a reduction in ESCC-associated mortality (2). However, ESCC eventually leads to inevitable locoregional recurrence and extensive metastasis. Therefore, there is a need to better understand the pathological and molecular features of ESCC, and to explore validated predictive biomarkers and novel treatment targets of the disease.

Previous studies have demonstrated that tumor immunogenicity, mediated by neoantigens, triggers an immune response in the host and provides immunological stimuli. Lymphatic subpopulations, therefore, preferentially traffic to the tumor mass and infiltrate spatially (3). Some studies indicated the presence of tumor-infiltrating lymphocytes (TILs) to be a favorable prognostic factor for survival in ESCC patients (4–6). However, TILs, also termed generalized TILs, are thought to be a heterogeneous group of lymphocytes possessing different, and even opposite, functions in anti-tumor activity (7). For instance, CD8+ TILs, also known as CD8+ cytotoxic T cells (CTLs), are directly capable of targeting tumor cells through binding with MHC class I molecules, and destroying tumor cells by releasing perforin or promoting apoptosis (8). A large amount of clinical data has proved that CD8+ TILs are associated with a better prognosis for ESCC patients (9–11). The role of CD4+ TILs differs depending on phenotype. Patients with abundant infiltration of CD4+ T helper type 1 lymphocytes (Th1) showed improved survival rates by stimulating CTLs, while CD4+ regulatory T cells (Tregs) are thought to inhibit effective anti-tumor response (12). Similarly, FoxP3+ Tregs are involved in maintaining immunological tolerance and suppressing effector T lymphocytes, and therefore predict an unfavorable prognosis (9). CD3+ TILs are one of the most representative TIL subtypes. Jesinghaus et al. found that high levels of intraepithelial CD3+ TILs were significantly associated with improved disease-free survival (DFS) and overall survival (OS) in ESCC (13), but other studies have reported opposite findings (12). CD45RO+ TILs have a helper induction effect, and studies have confirmed that CD45RO+ TILs could predict an improved DFS and OS, compared to negative patients (14). Furthermore, the pan-tumor spatial arrangement of TILs is a crucial predictive factor for recurrence and prognosis in non-small cell lung cancer (NSCLC) patients (15). Several studies have investigated the prognostic value of TILs based on their infiltrating location. To this end, we evaluated the prognostic efficacy of the different TIL subsets in ESCC, and determined the effect of their anatomical location. We hypothesized that the direction of prognostic influence of TILs would be similar in some subtypes, but that the magnitude of this effect might differ when considering the location of infiltration.

Published meta-analyses using widely differing methods have been conducted across many types of cancer, including melanoma, breast cancer, and many types of TILs (16, 17). Nevertheless, these studies show that there are definite and convincing conclusions linking TILs to prognosis in ESCC patients. To obtain a more precise estimate of prognostic value of TILs in ESCC patients, we carried out a comprehensive systematic review and meta-analysis for relevant publications.

## Methods

### Search Strategy

A literature search, based on title and abstract, was performed in PubMed/Medline, Embase, Web of Science and the Cochrane Library for articles using the following search strategy: (“tumor-infiltrating lymphocytes” OR TILs OR “T lymphocytes” OR “T cells” OR “Tregs”) AND (“Esophageal cancer”) (18). Two investigators (HAO and WANG) independently screened the titles and abstracts based on predetermined inclusion and exclusion criteria. Final inclusion was made following a full-text reading of the preliminary screenings. All discrepancies between the two researchers were discussed and resolved by consensus regarding the accuracy of inclusion. Additionally, reference lists of selected papers and related studies that were suggested by PubMed were also searched for potential missing articles. Finally, to avoid duplicates, two additional researchers reviewed all of the studies that were selected for inclusion. Advanced limitations were not imposed in the process of researching and selecting the articles.

### Inclusion and Exclusion Criteria

According to a previous study (19), reports that met the following criteria were deemed eligible for final inclusion: (1) prognostic value of generalized TILs and/or relevant subtypes were evaluated in patients with ESCC; (2) time-to-event survival analysis was incorporated with either DFS or OS to estimate the hazard ratios (HRs) and 95% confidence intervals (CIs); (3) Original articles were published in English between the prime and September 2018. Letters, reviews, case reports, animal trials, conference abstracts, clinical trials of immunotherapy, *in vitro* studies, and commentaries were excluded. To avoid publication bias that might exist in small studies, studies with n <30 patients were excluded.

### Data Extraction

Parameters were extracted from eligible publications using a predefined Microsoft Excel table, including the following fields: first author, year of publication, country, subtype, case number, location of infiltration, detection method, cut-off value for high or positive expression, tumor stage, follow-up time, and prognostic outcome of univariate and/or multivariate analysis (including HR, 95% CI, and *P*-value). When survival data were demonstrated by Kaplan-Meier curves rather than HRs, two researchers (HAO and WANG) then independently calculated data indirectly from the curves using Engauge Digitizer software (http://digitizer.sourceforge.net/) (20). When Kaplan-Meier curves were not available, or calculated HRs did not match the existing curves, studies were excluded. For time-to-event data, HRs were used to evaluate the risk of progression or death for patients with high-level TILs vs. low-level TILs. In studies that reported HRs for low-level TILs vs. high-level TILs, the reciprocals of HRs and 95% CIs were taken (19). Importantly, this meta-analysis extracted and processed survival data based on the cell type and infiltrating location, and classified the TILs as: pan-tumoral, also termed as entire tumor and general tumor, intra-tumoral (21, 22), also known as intraepithelial (6, 10, 13) and tumor nest (23), and peri-tumoral (21) or the tumor stroma (6, 11, 22).

### Quality Assessment

Selected publications were appraised to identify and assess any risk of biases that could be sufficiently large enough to distort the study results. The Quality In Prognosis Studies (QUIPS) handbook (24), which has been previously validated, was used to help reviewers who were conducting the systematic reviews. QUIPS is comprised of 6 bias domains, including study participation, study attrition, prognostic factor measurement, outcome measurement, study confounding, and statistical analysis and reporting. Each domain was rated as low, moderate, or high risk of bias by two researchers (HAO and WANG), independently. The reviewers' responses were considered, and any disagreement between two investigators was resolved through discussion. This meta-analysis also complied with the Preferred Reporting Items for Systematic Reviews and Meta-Analyses (PRISMA) guidelines (25).

### Statistical Analysis

As shown in Table 1, the included studies varied widely with regard to research methods. Therefore, a random-effects model was utilized when *I*^2^ > 50% or *P* < 0.1 to measure the heterogeneity of the studies. Otherwise, a fixed-effect model was applied. A quantitative measurement of inconsistency among studies was eventually demonstrated through visual inspection of forest plots. When heterogeneity was observed, sensitivity analysis was performed to test the stability of the main results. Additionally, asymmetry of a contour-enhanced funnel plot was used to evaluate the potential publication bias, and Begg's and Egger's tests were used to develop quantitative evidence. All analyses were completed by STATA version 12.0 (Stata Corporation, College Station, TX, USA), with significance defined as a *P*-value < 0.05 for overall results.

**Table 1:** Main characteristics of included studies.

**No**.	**Study**	**Country**	**Subsets**	**Case number**	**Location**	**Detected method**	**Cut-off value**	**Stage**	**Follow-up (M/Y)**	**Outcome**	**UA**	**MA**
1	Liu et al. (4)	China	TIL+	198	PA	IHC	Score = 11.19(UA), Grade3(MA)	I–III	m54M (6–152)	DFS	SC	Yes
2	Yasunaga et al. (26)	Japan	TIL+	78	PA	IHC, H&E	Score = 45.5(II), 54.3(III/IV)	II–IV	NR	OS	SC	Yes
3	Morita et al. (5)	Japan	TIL+	122	PA	H&E	Moderate(LI+)	I–IV	NR	OS	SC	No
4	Wang et al. (21)	China	CD8+	90	IT, PT	IHC	19/HPF(IT), 32/HPF(PT)	I–III	NR	DFS, OS	Report	No
5	Zhu et al. (27)^a^	China	CD8+	133	PA	IHC, H&E	61.44/HPF	IIb	m42.6M	DFS, OS	Report	No
			FOXP3+	133	PA	IHC, H&E	23.66/HPF	IIb	m42.6M	DFS, OS	Report	No
6	Chen et al. (12)	China	CD3+	514	IT, PT	IHC, H&E	Median count	I–IV	m32.7M (1–88.7)	DFS, OS	SC	Yes
			CD4+	530	IT, PT	IHC, H&E	Median count	I–IV	m32.7M (1–88.7)	DFS, OS	SC	Yes
			CD8+	527	IT, PT	IHC, H&E	Median count	I–IV	m32.7M (1–88.7)	DFS, OS	SC	Yes
7	Yoshioka et al. (8)	Japan	CD4+	122	PA	IHC	109/HPF	I–IVa	NR	OS	Report	Yes
			CD8+	122	PA	IHC	109/HPF	I–IVa	NR	OS	Report	Yes
			FOXP3+	122	PA	IHC	109/HPF	I–IVa	NR	OS	Report	Yes
8	Enomoto et al. (14)	Japan	CD45RO+	105	PA	IHC	113/HPF	I–IV	NR	OS	SC	Yes
9	Chen et al. (23)	China	CD3+	112	IT, PT	IHC	60/HPF	I–IV	NR	OS	Report	Yes
			CD8+	112	IT, PT	IHC	60/HPF	I–IV	NR	OS	Report	No
			FOXP3+	112	IT, PT	IHC	60/HPF	I–IV	NR	OS	Report	No
10	Li et al. (6)	China	TIL+	121	IT, PT	H&E	Score (range 0–90)	I–III	m34M (3–64)	DFS, OS	Report	Yes
11	Lv et al. (28)	China	TIL+	181	PA	IHC, IF	3.90/HPF	I–IV	NR	OS	Report	Yes
12	Hatogai et al. (12)	Japan	CD4+	196	PA	IHC, H&E	NR	I–IV	m5.5Y (0.1–10.6)	OS	Report	Yes
			CD8+	196	PA	IHC, H&E	NR	I–IV	m5.5Y (0.1–10.6)	OS	Report	Yes
			FOXP3+	196	PA	IHC, H&E	NR	I–IV	m5.5Y (0.1–10.6)	OS	Report	Yes
13	Jesinghaus et al. (13)	Germany	CD3+	125	IT	IHC, H&E	20 CD3is	I–IV	65.09M (mean)	OS	SC	Yes
14	Jiang et al. (22)	China	TIL+	235	IT, PT	H&E	20%(IT), 10%(PT)	I–IVa	m36M (30.0–42.1)	DFS, OS	Report	Yes
			CD4+	235	PT	IHC	10%	I–IVa	m36M (30.0–42.1)	DFS, OS	Report	No
			CD8+	235	IT, PT	IHC	10%	I–IVa	m36M (30.0–42.1)	DFS, OS	Report	No
			FOXP3+	235	IT, PT	IHC	10%	I–IVa	m36M (30.0–42.1)	DFS, OS	Report	No
15	Sugimura et al. (29)	Japan	CD8+	210	PA	IHC	15/HPF	NR	m35.1M	OS	Report	Yes
16	Zhu et al. (27)^b^	China	CD4+	220	PA	IHC	5%	NR	m53.25M	DFS, OS	Report	No
			CD8+	220	PA	IHC	16.90%	NR	m53.25M	DFS, OS	Report	No
			CD45RO+	220	PA	IHC	15.10%	NR	m53.25M	DFS, OS	Report	No
17	Tsuchikawa et al. (10)	Japan	CD4+	98	PA	IHC	1.4/HPF	I–IV	NR	OS	SC	No
			CD8+	98	PA	IHC	1.8/HPF	I–IV	NR	OS	SC	No
18	Zhang et al. (11)	China	CD8+	135	IT, PT	IHC, IB	10/HPF(IT), 20/HPF(PT)	I–IV	m49M (7–78)	OS	SC	No
19	Jiang et al. (31)	China	TIL+	250	PA	IHC, H&E	Score = 3	I–IV	m34.4M (0.3–147.1)	DFS, OS	Report	No

PA, pan-tumor; IT, intra-tumor; PT, peri-tumor; IHC, immunohistochemistry; H&E, hematoxylin-eosin staining; IF, immunofluorescence; IB, immunoblotting; LI, Lymphocyte infiltration; HPF, high power field; DFS, disease free survival; OS, overall survival; SC, survival curve; UA, univariate analysis; MA, multivariate analysis; NR, not report.

a 133 ESCC patients with pT3N0M0 stage who underwent radical Ivor-Lewis esophagetomies were include.

b *220 newly diagnosed ESCC patients of pT3N0M0 stage who had not undergone neoadjuvant therapy were included*.

## Results

### Study Selection and Characteristics

**Figure 1:**
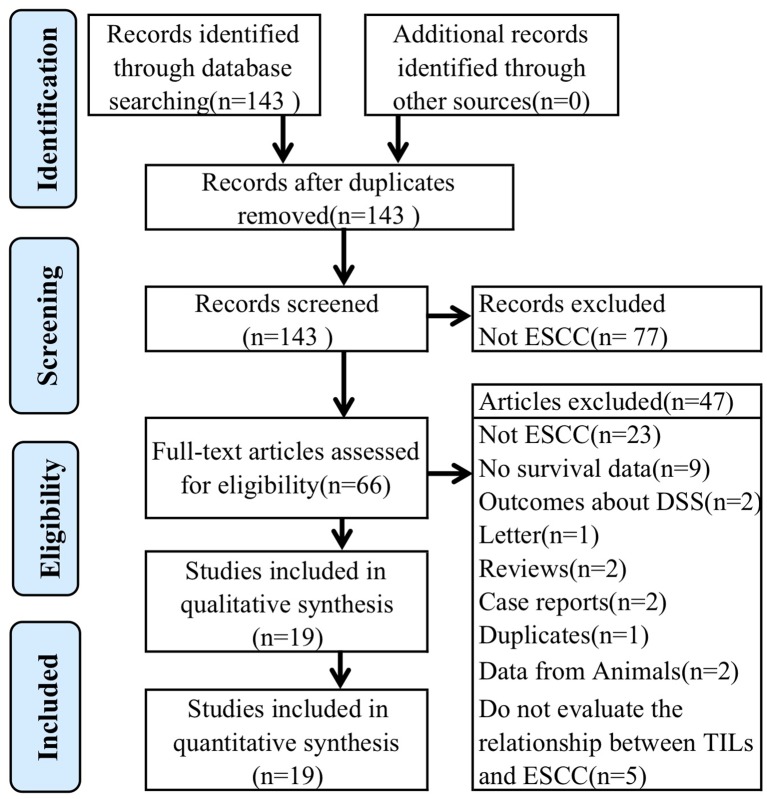
Flow diagram of study selection process.

After conducting literature search of the PubMed/Medline, Embase, Web of Science, and the Cochrane Library, we identified 143 potentially relevant articles (Figure 1). Following the screening of title and abstract, 77 articles were excluded because they were duplicates, not related to ESCC, or did not satisfy the inclusion criteria. Therefore, 66 full-text articles were evaluated, of which 19 met our inclusion criteria. The specific reasons for which these 47 studies were finally excluded are described in Figure 1. Table 1 summarizes some crucial characteristics of the 19 studies that were ultimately selected (4–6, 8–14, 21–23, 26–31). Only one study comes from a non-Asian country (13), which is consistent with the fact that Asians are susceptible to ESCC due to their hereditary backgrounds (32). Most studies investigated the prognostic value of at least two subgroups of TILs, and generalized TILs and CD8+ TILs were the most frequently studied. Only 3 studies have examined generalized TILs using H&E staining alone (5, 6, 31), and the remaining 16 studies recognized immunohistochemistry(IHC) as the dominate method for staining TILs. A common phenomenon shown in our meta-analysis is that the included studies have no universalized cut-off value for defining infiltrating lymphocyte levels. This issue generated the main research limitation. Additionally, 11 articles provided a median follow-up time, but did not provide details about people who were lost during follow-up.

### Results of Quality Assessment

QUIPS was used to appraise for risk of bias in the remaining 19 studies. In this systematic review and meta-analysis, almost no study presented detailed information about participants who were lost during follow-up, and most studies did not report any reasons for non-completion. This issue evidently leads to high risk of bias in the domain of study attrition. Unclear descriptions of cell counting methods and non-uniform cut-off values could provide other sources of bias in the domain of prognostic factor measurement. Factors that distorted the observed effect of TILs were classified in the study confounding domain. Indirect data from curves and diverse methodologies for analysis were not considered in the analysis and reporting domain (19). Low, moderate, and high risks of bias were reported in 3, 10, and 6 studies, respectively, with scores ranging from 1 to 6. As Hayden et al. recommended against the use of a summated score for overall quality, we did not exclude studies with high scores (24). The complete quality assessment of the publications is shown in Table 2.

**Table 2:** Quality assessment of included studies.

**No**.	**Study**	**Study participation**	**Study attrition**	**Prognostic factor**	**Outcome**	**Study con-founding**	**Analysis and reporting**		**Total risk of bias**
1	Liu et al. (4)	∘	•	∘	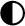	∘	∘	1	Low
2	Yasunaga et al. (26)	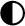	•	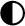	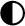	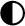	•	6	High
3	Morita et al. (5)	∘	•	∘	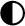	•	•	5	High
4	Wang et al. (21)	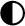	•	∘	∘	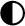	∘	2	Moderate
5	Zhu et al. (27)	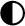	•	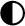	∘	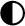	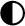	4	High
6	Chen et al. (12)	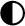	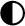	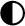	∘	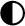	•	4	High
7	Yoshioka et al. (8)	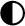	•	∘	∘	∘	∘	1	Moderate
8	Enomoto et al. (14)	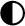	•	∘	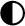	∘	∘	2	Moderate
9	Chen et al. (23)	∘	•	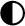	∘	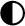	∘	2	Moderate
10	Li et al. (6)	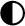	•	∘	∘	•	•	5	High
11	Lv et al. (28)	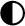	•	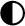	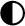	•	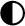	6	High
12	Hatogai et al. (12)	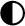	•	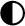	∘	∘	∘	2	Moderate
13	Jesinghaus et al. (13)	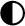	•	∘	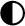	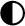	∘	3	Moderate
14	Jiang et al. (22)	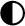	•	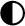	∘	∘	∘	2	Moderate
15	Sugimura et al. (29)	∘	•	∘	∘	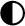	∘	1	Low
16	Zhu et al. (27)	∘	•	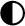	∘	∘	∘	1	Low
17	Tsuchikawa et al. (10)	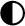	•	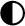	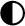	∘	∘	3	Moderate
18	Zhang et al. (11)	∘	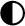	∘	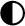	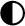	•	3	Moderate
19	Jiang et al. (31)	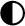	•	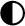	∘	∘	∘	2	Moderate

*∘, low risk of bias; 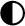, moderate risk of bias; •, high risk of bias*.

### Generalized TILs as Prognostic Biomarkers

**Figure 2:**
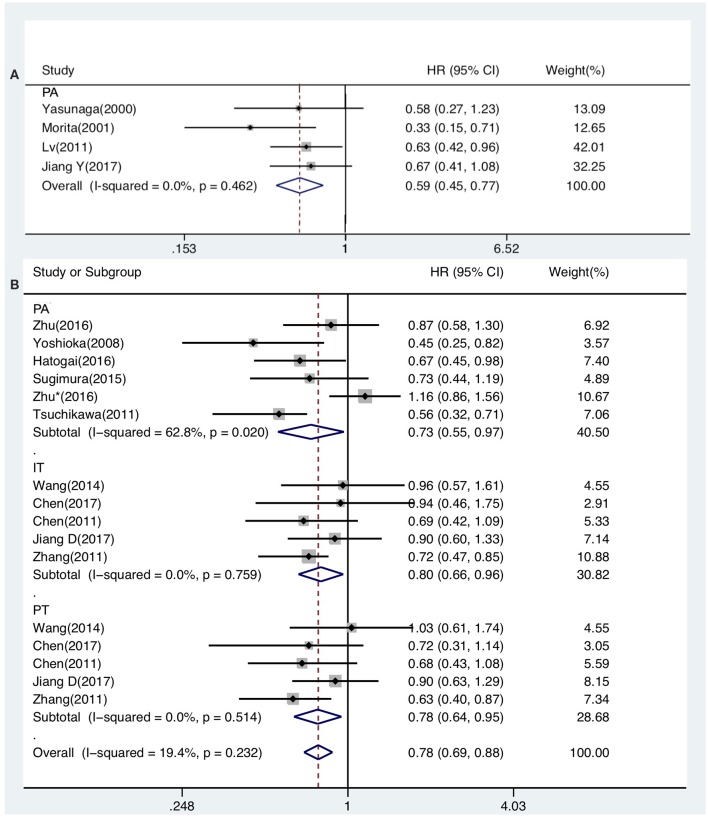
Forest plots of prognostic value of generalized TILs **(A)** and CD8+ TILs **(B)** on OS present on univariate analysis in ESCC patients.

**Figure 3:**
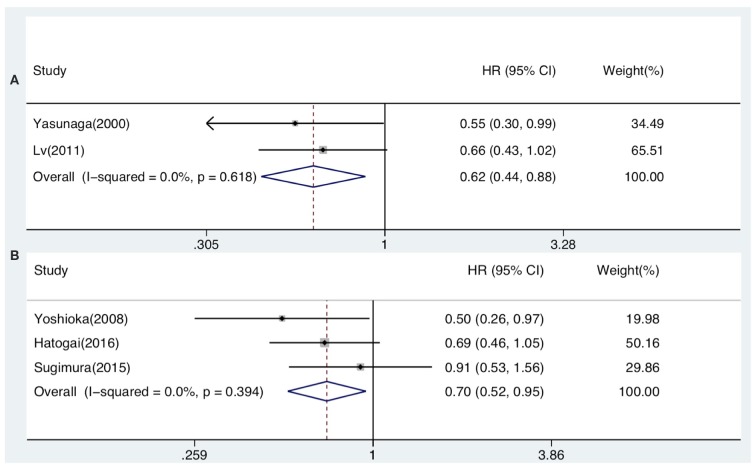
Forest plots of prognostic value of pan-tumor infiltration of generalized TILs **(A)** and CD8+ TILs **(B)** on OS present on multivariate analysis in ESCC patients.

The prognostic value of generalized TILs was assessed in 7 studies. The pooled analysis showed that both DFS [HR from univariate analysis: 0.630(0.415–0.955)] and OS [HR from univariate analysis: 0.586(0.447–0.770), HR from multivariate analysis: 0.621(0.439–0.878)] were positively associated with high-level infiltration of generalized TILs into the entire tumor masses. Nevertheless, pooled HRs from univariate analyses showed that high intratumor infiltration of generalized TILs did not correlate with DFS [HR: 0.774(0.414–1.445)] or OS [HR: 0.752(0.377–1.500)]. Similarly, high peritumor infiltration of generalized TILs was not associated with DFS [HR: 0.900(0.700–1.156)] or OS [HR: 0.860(0.632–1.170)] in univariate analysis, or in multivariate analysis [HR for DFS: 0.839(0.576–1.222); HR for OS: 0.793(0.505–1.245)] (Tables 3, 4, Figures 2, 3). In addition, one study that was excluded found a significant influence of generalized TILs on cancer-specific survival in patients with pan-tumor infiltration (33).

**Table 3:** The pooled univariate analysis of generalized TILs and subsets.

**Subset**	**Outcome**	**Location**	**Study number**	**Case number**	**HR(95%CI)**	***P*-Value**	**Heterogeneity**		**Begg's test**		**Egger's test *P*-Value**
							***I*2, %**	***P*-value**	**Z**	***P*-Value**	
TIL+	DFS	PA	2	448	0.630 (0.415–0.955)	0.029	20.1	0.263	0.000	1.000	–
		IT	2	356	0.774 (0.414–1.445)	0.421	72.9	0.055	0.000	1.000	–
		PT	2	356	0.900 (0.700–1.156)	0.409	61.6	0.107	0.000	1.000	–
	OS	PA	4	631	0.586 (0.447–0.770)	0.000	0.0	0.462	1.020	0.308	0.256
		IT	2	356	0.752 (0.377–1.500)	0.418	75.0	0.045	0.000	1.000	–
		PT	2	356	0.860 (0.632–1.170)	0.335	71.9	0.059	0.000	1.000	–
CD8+	DFS	PA	2	353	1.026 (0.814–1.292)	0.830	0.0	0.534	0.000	1.000	–
		IT	3	852	0.901 (0.678–1.198)	0.474	0.0	0.943	1.040	0.296	0.021
		PT	3	852	0.949 (0.730–1.233)	0.693	0.0	0.998	1.040	0.296	0.362
	OS	PA	6	979	0.733 (0.555–0.968)	0.028	62.8	0.020	1.130	0.260	0.064
		IT	5	1,099	0.797 (0.660–0.962)	0.018	0.0	0.759	0.730	0.462	0.288
		PT	5	1,099	0.776 (0.635–0.948)	0.013	0.0	0.514	0.240	0.806	0.940
CD4+	DFS	PT	2	765	0.857 (0.463–1.585)	0.623	0.0	0.630	0.000	1.000	–
	OS	PA	4	636	0.726 (0.480–1.097)	0.129	70.2	0.018	1.020	0.308	0.201
		PT	2	765	0.757 (0.397–1.446)	0.400	0.0	0.690	0.000	1.000	–
FOXP3+	OS	PA	3	451	0.920 (0.489–1.731)	0.796	82.5	0.003	0.000	1.000	0.074
		IT	2	347	0.880 (0.245–3.164)	0.845	49.8	0.158	0.000	1.000	–
		PT	2	347	1.367 (0.884-2.115)	0.160	0.0	0.933	0.000	1.000	–
CD3+	OS	IT	3	751	0.678 (0.380–1.208)	0.187	60.5	0.079	1.040	0.296	0.623
		PT	2	626	0.867 (0.407–1.847)	0.712	80.3	0.024	0.000	1.000	–
CD45RO+	OS	PA	2	325	0.652 (0.273–1.554)	0.334	74.1	0.050	0.000	1.000	–

*HR, hazard ratio; CI, confidence interval*.

**Table 4:** The pooled multivariate analysis of generalized TILs and subsets.

**Subset**	**Outcome**	**Location**	**Study number**	**Case number**			**Heterogeneity**		**Begg's test**		**Egger's Test *P*-value**
					**HR(95%CI)**	***P*-Value**	***I*2, %**	***P*-value**	**Z**	***P*-Value**	
TIL+	DFS	PT	2	356	0.839 (0.576–1.222)	0.361	80.3	0.024	0.000	1.000	–
	OS	PA	2	259	0.621 (0.439–0.878)	0.007	0.0	0.618	0.000	1.000	–
		PT	2	356	0.793 (0.505–1.245)	0.313	85.8	0.008	0.000	1.000	–
CD8+	OS	PA	3	528	0.705 (0.524–0.947)	0.020	0.0	0.394	0.000	1.000	0.764
CD4+	OS	PA	2	318	0.785 (0.552–1.116)	0.177	0.0	0.978	0.000	1.000	–
FOXP3+	OS	PA	2	318	0.776 (0.280–2.151)	0.626	85.8	0.008	0.000	1.000	–
CD3+	OS	IT	3	751	0.958 (0.498–1.842)	0.898	74.6	0.019	0.000	1.000	0.647
		PT	2	626	1.205 (0.860–1.688)	0.278	0.0	0.795	0.000	1.000	–

### CD8+ TILs as Prognostic Biomarkers

A total of 12 articles investigated the prognostic value of CD8+ TILs in patients with ESCC. Pooled HRs showed that patients with high level of CD8+ TILs in the entire tumor mass had an unfavorable outcome for DFS [HR from univariate analysis: 1.026(0.814–1.292)], but a favorable outcome for OS [HR from univariate analysis: 0.733(0.555–0.968), HR from multivariate analysis: 0.705(0.524–0.947)]. Pooled HRs from univariate analysis in patients with intratumor infiltration showed that high level of CD8+ TILs was not correlated with DFS [HR: 0.901(0.678–1.198)], but was associated with a favorable outcome for OS [HR: 0.797(0.660–0.962)]. Similarly, pooled HRs from univariate analysis showed that high peritumor infiltration of CD8+ TILs was not correlated with DFS [HR: 0.949(0.730–1.233)], but was associated with a favorable outcome for OS [HR: 0.776(0.635–0.948) (Table 3, Figures 2, 3).

### CD4+ TILs as Prognostic Biomarkers

Six studies presented data on the prognostic value of CD4+ TILs. The pooled HRs showed that high levels of CD4+ TILs infiltration in the entire tumor mass was not correlated with OS [HR from univariate analysis: 0.726(0.480–1.097), HR from multivariate analysis: 0.785(0.552–1.116)]. Meanwhile, pooled HRs from univariate analyses indicated that high peritumor infiltration of CD4+ TILs was not related to DFS [HR: 0.857(0.463–1.585)] or OS [HR: 0.757(0.397–1.446)] (Tables 3, 4).

### FoxP3+ TILs as Prognostic Biomarkers

Five included studies demonstrated the prognostic value of FoxP3+ TILs on OS. No correlation was observed between high levels of FoxP3+ TILs infiltration in whole tumor mass and OS [HR from univariate analysis: 0.920(0.489–1.731), HR from multivariate analysis: 0.776(0.280–2.151)]. Similarly, high intratumor and peritumor infiltration of FoxP3+ TILs was not correlated with OS. The pooled HRs from univariate analyses were 0.880(0.245–3.164) and 1.367(0.884–2.115) for intratumor and peritumor infiltration, respectively (Tables 3, 4).

### CD3+ TILs as Prognostic Biomarkers

Three studies that evaluated the impact of CD3+ TILs on survival were included in this meta-analysis. The studies investigating CD3+ TILs only explored the relationship between infiltration level and OS. High levels of intratumor CD3+ TILs did not improve patient survival. The pooled HRs of OS from univariate and multivariate analysis were 0.678(0.380–1.208) and 0.958(0.498–1.842), respectively. Additionally, the pooled HR from univariate analysis showed that high peritumor infiltration of CD3+ TILs did not correlate with OS [HR: 0.867(0.407–1.847)] However, the pooled HR from multivariate analysis showed a contradictory result [HR: 1.205(0.860–1.688)] (Tables 3, 4).

### CD45RO+ TILs as Prognostic Biomarkers

Only 2 studies evaluated the impact of CD45RO+ TILs on OS, and only one study (34), which had been excluded, reported data on DFS. Therefore, there was little available data to use to determine the effect of CD45RO+ TILs on OS in patients. The pooled HR from univariate analysis was 0.652(0.273–1.554), indicating that no correlation was found, and that further research is urgently needed (Table 3).

### Sensitivity Analyses and Publication Bias

Sensitivity analyses were performed to assess the stability of the results based on a published study (35). No individual study changed the pooled data qualitatively according to the leave-one-out trial. Simultaneously, the profile of the whole funnel plots appeared to be symmetrical, indicating the absence of publication bias. Additionally, the results of Begg's test and Egger's test showed no significant publication biases that could have significantly influenced the results. We, therefore, did not use the non-parametric “trim-and-fill” method.

## Discussion

This study evaluated the prognostic value of TILs as a reasoned method to explore prognostic markers for ESCC patients. However, retrospective studies conducted on TILs have shown incompatible conclusions regarding the prognostic efficacy of TILs. We, therefore, systematically reviewed and analyzed previous studies to determine the correlation between TILs and the clinical outcomes of ESCC patients.

First, we investigated the prognostic effect of generalized TILs by meta-analysis. The result indicated that generalized TILs that infiltrate the entire tumor mass are associated with a favorable prognosis for DFS and OS. This finding was consistent with the hypothesis that dense lymphatic infiltration in primary tumors would be associated with improved survival rates in breast cancer (17), melanoma (16), and ovarian cancer (36). Conversely, intratumor and peritumor infiltration of generalized TILs was not correlated with survival outcome in this meta-analysis. Our analyses demonstrated the prognostic value of generalized TILs, however understanding the spatial organization of generalized TILs within the whole tumor mass would improve the predictive value of TILs for cancer prognosis (15). Additionally, local density of TILs, subpopulations, and colocalization of cancerous cells may differ depending on the spatial architecture of TILs (37). Therefore, the location and extent of infiltration would be the predominant factors that influence the prognostic value of generalized TILs.

However, some studies reported that abundant TILs were not related to prognosis in ESCC patients, regardless of infiltrating location (26, 31). Differences in clinical methodology could explain these divergent conclusions. For instance, when clinical stage, tumor grade, and follow-up time were taken into account, TILs can improve outcome prediction even more significantly (38). Since advanced stage and higher grade tumors usually harbor more differentiated TILs, the latter may gradually lose the ability to induce tumor regression due to downregulation of lymphoid homing (CD62L) and costimulatory (CD27 and CD28) molecules (39). This means that more differentiated TILs might have a lower anti-tumor activity. In addition, patient cohorts in the included studies received diverse treatment modalities, such as chemoradiotherapy, which would increase the tumor's response to TILs (40). The underlying mechanisms of the interaction, such as improved response to cytokines, have not yet been fully elucidated.

Considering the great heterogeneity of TILs, the phenotype of infiltrating lymphocytes varies greatly. CD8+ TILs are the most frequently assessed subtype as they are considered the pivotal effector of the immune system against malignancies. Many studies have found a favorable survival outcome for patients with high-levels of infiltration of CD8+ TILs (8–11). In accordance with these findings, our study found that patients with abundant CD8+ TILs had better OS than those with a lower infiltration. This result was not influenced by the location of TILs infiltration. On the other hand, some studies did not observe any prognostic significance of CD8+ TILs (12, 21, 27). This discrepancy may be explained by adoptive immune-resistance, induced by the upregulation of PD-L1 or PD-1, which suppresses the immune activity of the tumor microenvironment (41). Similarly, Chen et al. showed that the expression of B7-H4 on esophageal cancer cells is inversely correlated with the densities of CD8+ TILs, suggesting that B7-H4 inhibits TILs recruitment (23). In addition, the impaired function of CD8+ TILs may also be mediated by immunosuppressing factors released by tumor cells, deficient presentation of tumor antigen by dendritic cells, and reduced production of co-stimulating cytokines by helper CD4+ T-cells (42, 43).

CD4+ TILs play an immunomodulatory role in the host immune system (44). The proportion of CD4+ and CD8+ TILs reflects changes in the body's immune system, and differs among patients and infiltrating locations (45). The results of this meta-analysis suggested that CD4+ TILs are not associated with survival outcome. However, previous studies observed that ovarian cancer patients with increased intratumor infiltration of CD4+ lymphocytes had longer OS (46). Considering the ambiguous role of CD4+ lymphocytes in ESCC, and the small number of eligible studies, we conclude that the prognostic role of CD4+ TILs remains questionable. Furthermore, subpopulations of CD4+ TILs could have different functional impacts on the immune environment (44). For instance, this study showed that high infiltration of FoxP3+ regulatory subset had no prognostic value for ESCC. However, previous findings demonstrated that high FoxP3+ Tregs density was associated with a significant lower OS rate in melanomas (47) and hepatocellular carcinoma (48). Foxp3 is considered the most specific Tregs marker, however it is not specific for activated Tregs. Additional markers, such as CD25 and CD127, might be required to determine the immunological functions of Tregs.

In this meta-analysis, we investigated the prognostic value of CD3+ TILs, which are generally considered to be a positive predictor of prognosis for ESCC patients. In clinicopathological practice, CD3 is one of the most representative molecules used to assess the overall quantity of infiltrating T lymphocytes. In the tumor microenvironment, CD3 could be used to determine whether a given cancer can be considered to be in the state of a “T-cell inflamed microenvironment” (49). Nevertheless, the pooled analysis found that infiltration of CD3+ TILs was not correlated with prognosis. This result does not correspond with previous studies that show that CD3+ TILs play an important role in antitumor activity. Jesinghaus et al. suggested that abundant infiltration of intraepithelial CD3+ TILs is associated with favorable survival outcomes in ESCC patients (13). Therefore, detecting intraepithelial infiltration of CD3+ TILs is a comparatively specific method for predicting tumor prognosis. Because intraepithelial lymphocytes are more likely to interact with cancer cells than their stromal counterparts, the localization of TILs seems to have major relevance with regards to their prognostic impact.

CD45RO has been generally accepted as the optimal single marker for the entire memory T cell population, with the exception of T memory stem cells (50). To the best of our knowledge, few studies have previously addressed the role of CD45RO+ TILs in ESCC. These T cells include both CD4+ and CD8+ T lymphocytes that have been exposed to antigens, which are known to respond faster than naïve T cells upon re-stimulation with antigens (51). In our study, CD45RO+ TILs were not associated with patients' survival outcome, which could be attributed to the overexpression of immunosuppressive cytokines such as IL-10 and TGF-β, which are known to effectively inhibit the function of the CTLs (52). However, previous studies have reported that high density of CD45RO+ T memory cells is associated with better disease-related outcomes in various human cancers, including EAC and ESCC (47). Some data also suggest that CD45RO+ T cells generated in the primary tumor may have the ability to control micrometastatic cancer cells in lymph nodes or distant organs in the post-operative period (14). Further studies are needed to confirm the prognostic role of CD45O+ TILs in ESCC patients.

The checks and balances between subpopulations can also influence the immunocompetence of TILs. These reciprocal interactions may be verified by the ratio of CD8+ TILs to other subtypes. For instance, in combination with CD8+ TILs, Foxp3+ TILs could also be used as a prognostic indicator. A higher CD8+/Foxp3+TILs ratio, which indicates that the beneficial effect of CD8+ T cells outweighs the immunosuppressive effect of the Tregs, was a better indicator for survival outcome than CD8+ or Foxp3+ TILs alone (9, 27). In addition, a suitable CD8+/CD4+ TILs ratio corresponds with a favorable prognosis and reflects the immune response against ESCC (38). Therefore, the balance of CD8+ and CD4+ TILs is critical for the prognosis of patients with ESCC. However, an insufficient number of studies that evaluate these ratios were available in this meta-analysis. Therefore, future research should be conducted to develop a more comprehensive understanding of the value of using these ratios as a prognostic marker.

Tumor microenvironment plays a pivotal role in the anti-cancer immunity of TILs. The programmed cell death protein 1 (PD-1) is an immunoinhibitory receptor expressed on activated CD4+ and CD8+ TILs that, together with its ligands PD-L1 and PD-L2 that are expressed in tumor cells, helps to negatively regulate TILs activation (53, 54). Preclinical data indicated that PD-1+ TILs displayed an impaired effector function to proliferate and produce cytokines, thus termed functional exhaustion. This finding provided a plausible explanation for tumor progression despite the presence of TILs (55). Yagi et al. (56) reported that patients with PD-L1+ esophageal cancer cells significantly associated with worse OS (HR, 1.69; 95% 1.05–2.67; *P* < 0.033), compared with PD-L1 negative cases. Similarly, Chen et al. (23) revealed that levels of PD-L1 expression on esophageal cancer cells were inversely correlated with the density of CD3+ and CD8+ TILs, indicating a possible role of PD-L1 in suppressing immune surveillance. In consequence, tumors with PD-1+ or PD-L1+ TILs are most likely to benefit from a single-agent anti-PD-1/PD-L1 blockade, as these tumors possess pre-existing TILs that are turned off by PD-1/PD-L1 engagement. Kudo et al. (57) demonstrated that nivolumab, a monoclonal antibody specific for PD-1, showed promising anti-cancer activity in patients with ESCC. However, when combined PD-1/PD-L1 expression with TILs status, the prognostic efficacy will be different from the single marker. Preclinical data revealed that ESCC patients who had PD-L1+ TILs were significantly associated with improved OS (HR, 2.01; 95% 1.14–3.41; *P* < 0.0001) and had lower risk of distant recurrence (42.1 vs. 72.3%; *P* = 0.042), using PD-L1- TILs as a referent (56, 58). In this meta-analysis, we could not merge the HRs of PD-1+ or PD-L1+ TILs due to insufficient data. Taken together, PD-1+ or PD-L1+ TILs may be a promising biomarker for identifying patients who may benefit from immune-checkpoint inhibitors. In the future, more studies that investigate the intercorrelation between PD-1/PD-L1 pathway and TILs in ESCC, are imperiously needed.

Our meta-analysis has certain limitations that are inherent to its design, and to features of the included articles. First, the main limitation was the heterogeneous study cohorts. Patient cohorts included in this study have different case numbers, clinical stages, pathological stages, and follow-up times, which influence the prognostic value of the biomarkers through different mechanisms. Very few studies accounted for treatment modality in their analysis, but the given therapies also influence immune status via immunological mechanisms. Therefore, to strengthen the prognostic value of TILs, they should be studied in homogenous cohorts. Second, the determined cutoff points differed widely among the included studies. Some studies use percentiles, tertiles, or the median, whereas others use absence vs. presence, the minimal *P*-value approach, or do not report a cutoff point at all. Therefore, it is difficult to determine the precise quantification of the TILs. To incorporate TILs in clinical practice, it is necessary to establish a standardized and validated cut-off point for quantification. However, it was not yet possible to suggest a universally applicable cut-off point in this meta-analysis. Third, Foxp3, CD4, and CD8 are not exclusively representative of T helper cells and cytotoxc T-cells. They are also observed on macrophages and dendritic cells. Modern techniques that are able to identify T-cell subsets more specifically might provide more robust biomarkers for ESCC. Fourth, all of the included studies were retrospective. Currently, technical issues related to producing tumor-specific T cells present a formidable barrier to conducting randomized clinical trials. These limitations raise the question of whether biology or methodology is the source of the observed prognostic effects of TILs for survival. Biological support can be gained from *ex vivo* and *in vitro* studies, which offer a more detailed perspective. Scientific design can be one of the methodological supports to reduce such limitations.

In conclusion, our meta-analysis confirmed the prognostic role of both generalized TILs and CD8+ TILs in ESCC. High-level infiltration of generalized and CD8+ TILs predicted a better OS, for death from all causes. In order to incorporate prognostic T-cell markers into clinical practice, more prognostic studies with homogeneous patient cohorts, with respect to infiltrating location, tumor stage, and treatment modality, are needed.

## Data Availability Statement

All datasets generated for this study are included in the article/supplementary material.

## Author Contributions

JH, SW contributed to the conception and design of the study. ML, TZ, HY, YL, and YX organized the databases and provided methodological supports. JH, ML, and SW performed the statistical analysis. JH wrote the draft of the manuscript. YX, RA, and SW contributed to the supervision of the study. All authors critically reviewed and revised the manuscript for important intellectual content and approved the final version of the manuscript before submission.

## Conflict of Interest

The authors declare that the research was conducted in the absence of any commercial or financial relationships that could be construed as a potential conflict of interest.
